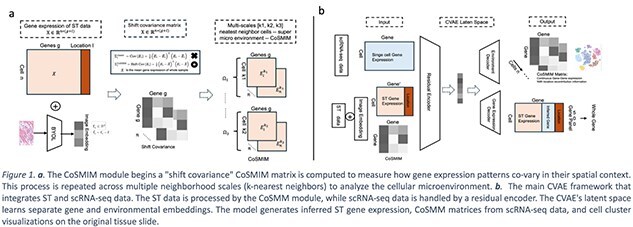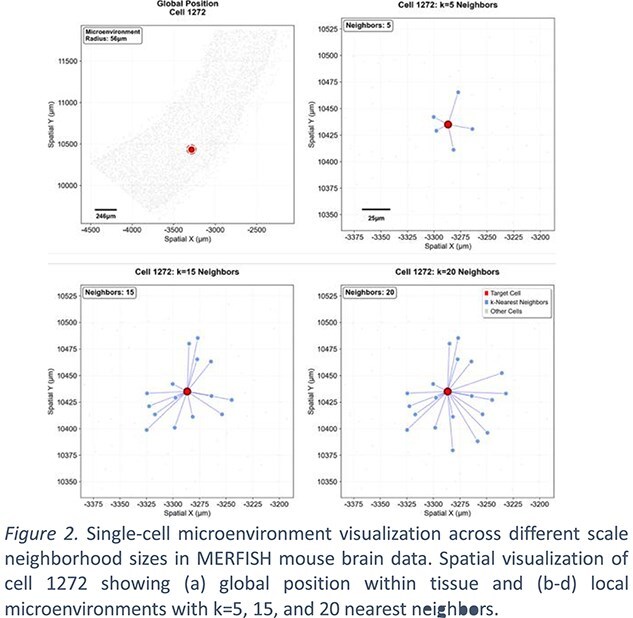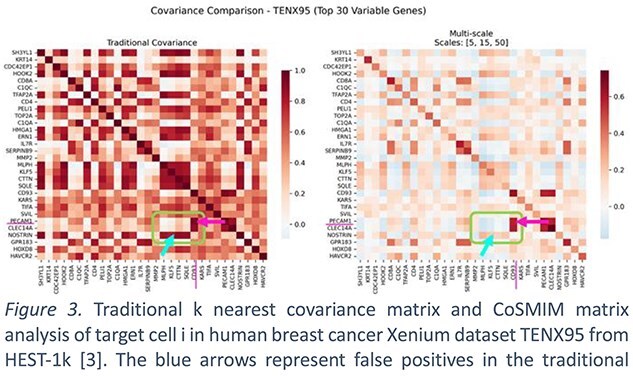# CoSMIM: a covariance-based multi-scale framework for integrated spatial microenvironment analysis

**DOI:** 10.1093/bib/bbaf631.003

**Published:** 2025-12-12

**Authors:** Bairui Du, Hisanori Kiryu

**Affiliations:** Department of Computational Biology and Medical Sciences, Graduate School of Frontier Sciences, The University of Tokyo; Department of Computational Biology and Medical Sciences, Graduate School of Frontier Sciences, The University of Tokyo

## Abstract

**Introduction:**

The tumor microenvironment (TME) is a complex ecosystem where cellular interactions are spatially governed. Spatial transcriptomics (ST) technologies, sequencing-based Visium which only have spot level resolution and imaging-based Xenium which are limited by a small RNA panel, probe the complexity, but high dimensional data interpretation remains a challenge [1].

Current analyses rely on pre-defined cell annotation patterns, critically failing to account for higher-order structures in gene co-expression and cell co-localization [2]. Biologically, cellular function is defined by coordinated gene modules, not individual genes, and these relationships exhibit a hierarchical organization across multiple spatial scales. A simple gene expression matrix is insufficient to capture this multi-scale and hierarchical structure of gene–gene and cell–cell interactions, especially given that TME niches are highly variable and context-dependent, difficultly to make a fixed scale definition. Furthermore, the order of cells in discrete gene expression matrices can affect the ability of neural networks to analyze cell–cell interactions.

To overcome this, we introduce **CoSMIM (Covariance of Spatial Multi-scale Integrated Microenvironment)** matrix. Our hypothesis is that gene–gene covariance matrices, computed across multiple scales of neighbor cells, provide a richer representation of the TME’s spatial heterogeneity functional architecture. We use a multi-decoder conditional variational autoencoder(CVAE) to learn a unified latent space for consistent cross-scale modeling. Its architecture can impute genes in targeted spatial panels and infer spatial covariance matrices for non-spatial single-cell data, thus bridging these modalities.

**Methodology:**

Our framework consists of two core components: the construction of a CoSMIM niche representation and a CVAE generative model for learning a unified representation. The input to our method is ST data from dataset HEST-1 k and 10X human-breast cancer FFPE dataset [3,4] which has various ST platforms such as Visium, Xenium, CosMx, or MERFISH. The specific steps are as follows (Fig. 1b):

**Definition and construction of multi-scale neighborhoods:**

Tumor spatial heterogeneity is not random but is driven by the organizational patterns of cellular communities at different scales. Precisely defining cellular niches within ST data remains a key challenge. Given the heterogeneity in cell size, defining neighborhoods using a fixed number of cells or a constant radius has limitations [5]. We propose an adaptive approach that constructs neighborhoods through a learned linear combination of different biological scales (Fig. 2&Eq.2). We predefine a series of spatial neighborhood representing distinct biological interaction ranges.

The gene expression matrix of whole tissue is $\mathrm{X}\in{R}^{n\times g}$, $n$ is the number of cells and $\mathrm{g}$ is number of genes. Each cell has a muti-scale niches matrix ${E}_k^i\in{R}^{k\times g}\left(\mathrm{e}.\mathrm{g}.\mathrm{k}\in \mathrm{K}\left\{\mathrm{5,15,50}\right\}\right)$ of $k$ nearest neighbor cells (excluding the target cell $i$ itself). Let $\mathrm{Cov}\in{\mathrm{R}}^{g\times g}$ be the target cell $i$’s gene–gene covariance of $k$ nearest neighbor cells. $\overline{X}$ global mean gene expression of gene g and $\overline{E_k}$ is $k$ scale niche’s mean gene expression.

$$ \mathrm{Co}{v}_k^{troditional}={\sum}_{i=1}^n Co{v}_k\left({E}_i\right)={\sum}_{i=1}^n\frac{1}{k}{\left({E}_i^k-\overline{E_i^k}\right)}^T\left({E}_i^k-\overline{E_i^k}\right) $$

$$ \mathrm{Co}{v}_k^{our\; method}={\sum}_{i=1}^n\mathrm{Co}{\mathrm{v}}_{\mathrm{k}}\left({E}_i^k\right)={\sum}_{i=1}^n\frac{1}{k}{\left({E}_i^k-\overline{X}\right)}^T\left({E}_i^k-\overline{X}\right) $$

$$ CoSMIM=\sum_{k={k}_1}^K{w}_k Co{v}_k^{ourmethod},{w}_k\ge 0,\sum_{k={k}_1}^K{w}_k=1(1) $$

We then evaluate and optimize the selection of these scales $K$ based on the discriminatory power of their corresponding eigenvalues in principal component analysis. These scales correspond to different biological contexts:

**Small-scale (5–15 cells):**

Primarily captures direct signaling and short-range paracrine signaling, such as direct contact between a tumor cell and an adjacent immune cell PD-1/PD-L1 or local growth factor support from a Cancer-Associated Fibroblast.

**Medium-scale (15–50 cells):**

Reflects the characteristics of a functional niche, such as an immunosuppressive or pro-angiogenic ‘community’ composed of several tumor, immune, and stromal cells.

**Large-scale (>$50cells):**

Characterizes the tissue region state, such as broad hypoxic zones, regions of lymphocytic infiltration, or fibrotic stromal areas, which are often governed by diffusive chemical gradients or physical barriers.

The scale weights are learned by entropy-regularized log-det minimization:

$$ {\mathrm{w}}^{\ast }=\arg \underset{\mathrm{w}\in{\Delta}^{\mathrm{K}-1}}{\min}\left(-\log \left|\Sigma \left(\mathrm{w}\right)+\upvarepsilon \mathrm{I}\right|+\uplambda{\sum}_{\mathrm{k}=1}^{\mathrm{K}}{\mathrm{w}}_{\mathrm{k}}\log{\mathrm{w}}_{\mathrm{k}}\right)\kern0.36em (2) $$

With $\varepsilon >0$ for numerical stability and $\lambda \ge 0$ (larger $\lambda \Rightarrow$ more uniform $w$; smaller $\lambda \Rightarrow$ more concentrated $w$).

The weighted CoSMIM matrix can be conceptualized as a ‘multi-scale spatial fingerprint’ of a niche. Using the progression of breast cancer from Ductal Carcinoma in Situ (DCIS) to Invasive Ductal Carcinoma (IDC) as an example, we hypothesize that this spatial fingerprint undergoes a specific transformation. In the DCIS stage, cancer cells are confined within the duct. Their microenvironmental communication is dominated by small-scale interactions, and thus the CoSMIM-learned weights are likely biased towards smaller scales.

Upon progression to IDC, cancer cells breach the basement membrane, initiating large-scale invasion, remodeling the surrounding stroma, inducing angiogenesis, and interacting with broader immune populations, leading to infiltration and fibrosis. At this stage, medium and large-scale interactions become critically important. Therefore, this learned and quantifiable shift in scale weights has the potential to serve as an early biomarker for assessing invasion risk in breast cancer.

Traditional cell/spot-gene expression matrices are sensitive to the ordering of cells, which impacts model robustness. We employ a feature representation that is invariant to cell permutation, the gene–gene correlation covariance matrix.

**Beyond Ligand-Receptor Pair Analysis:**

Conventional Ligand-Receptor (L-R) analysis often focuses on the co-expression of a few L-R pairs, overlooking downstream signaling cascades. The covariance matrix excels at capturing the holistic activation state of an entire ‘signaling module.’

**Capturing Long- and Short-Range Signaling:**

When calculating the CoSMIM covariance, we use the global mean gene expression as the centering baseline [Fig. 1a], rather than the conventional local neighborhood mean. This design enables CoSMIM to not only capture local signaling patterns reflecting autocrine, paracrine, and juxtacrine modes but also to identify long-range endocrine signaling between spatially distant cells in a tissue sample.

**Result and conclusion:**

In a direct comparison using human breast cancer transcriptomes, our CoSMIM framework demonstrated superior accuracy over traditional covariance methods in defining the spatial interactome (Fig. 3). While both approaches pinpointed the well-established endothelial–immune axis, marked by strong PECAM1–CD93 and CLEC14A–CD93 co-expression, only CoSMIM could confirm that this interaction was a fundamental architectural feature, persisting across all spatial scales. This finding validates CD93 as a stable regulator of the angiogenic and immune-suppressive barrier. Traditional covariance analysis once suggested a highly correlated expression network linking NOSTRIN with genes involved in matrix remodeling (MMP2), cell proliferation (KLF5), and metabolism (SQLE) (Fig. 3). However, CoSMIM revealed that this apparent high correlation was likely a false-positive signal driven by spatial heterogeneity, and no strong correlation exists within its multiscale spatial niche.

By confirming genuine interactions while systematically eliminating such spatial artifacts, CoSMIM offers a profoundly clearer view of the breast cancer microenvironment. It moves beyond simple co-expression to identify mechanistically robust signaling axes, providing a high-fidelity roadmap for prioritizing novel therapeutic targets.

**References:**

1. Haviv D., Remšík J., Gatie E.A.A. et al. ‘The Covariance Environment Defines Cellular Niches for Spatial Inference.’ Nature Biotechnology 2025;43:269–280.

2. Kojima Y. et al. ‘Single-Cell Colocalization Analysis Using a Deep Generative Model.’ Cell Systems 2024;15:180–192.e7.

3. Jaume G. et al. ‘HEST-1 k: A Dataset for Spatial Transcriptomics and Histology Image Analysis.’ arXiv 2024;arXiv:2406.16192.

4. Janesick A. et al. ‘High resolution mapping of the tumor microenvironment using integrated single-cell, spatial and in situ analysis.’ Nature Communications 2023;14:8353

5. Yang Y. et al. ‘STAIG: Spatial transcriptomics analysis via image-aided graph contrastive learning for domain exploration and alignment-free integration.’ Nature Communications 2025;16:1067.